# Early post-mortem formation of carbonate concretions around tusk-shells over week-month timescales

**DOI:** 10.1038/srep14123

**Published:** 2015-09-15

**Authors:** Hidekazu Yoshida, Atsushi Ujihara, Masayo Minami, Yoshihiro Asahara, Nagayoshi Katsuta, Koshi Yamamoto, Sin-iti Sirono, Ippei Maruyama, Shoji Nishimoto, Richard Metcalfe

**Affiliations:** 1 Material Research Section, Nagoya University, University Museum, Chikusa, Nagoya, Japan; 2 Graduate School of Environmental Studies, Nagoya University, Chikusa, Nagoya, Japan; 3 Center for Chronological Research, Nagoya University, Chikusa, Nagoya, Japan; 4 Department of Education, Gifu University, Gifu, Japan; 5 Nagoya City Science Museum, Sakae, Nagoya, Japan; 6 Quintessa UK, The Hub, Henley-on-Thames, Oxfordshire, UK

## Abstract

Carbonate concretions occur in sedimentary rocks of widely varying geological ages throughout the world. Many of these concretions are isolated spheres, centered on fossils. The formation of such concretions has been variously explained by diffusion of inorganic carbon and organic matter in buried marine sediments. However, details of the syn-depositional chemical processes by which the isolated spherical shape developed and the associated carbon sources are little known. Here we present evidence that spherical carbonate concretions (diameters φ : 14 ~ 37 mm) around tusk-shells (*Fissidentalium* spp.) were formed within weeks or months following death of the organism by the seepage of fatty acid from decaying soft body tissues. Characteristic concentrations of carbonate around the mouth of a tusk-shell reveal very rapid formation during the decay of organic matter from the tusk-shell. Available observations and geochemical evidence have enabled us to construct a ‘Diffusion-growth rate cross-plot’ that can be used to estimate the growth rate of all kinds of isolated spherical carbonate concretions identified in marine formations. Results shown here suggest that isolated spherical concretions that are not associated with fossils might also be formed from carbon sourced in the decaying soft body tissues of non-skeletal organisms with otherwise low preservation potential.

Isolated spherical carbonate concretions in sedimentary rocks are localized volumes that are highly enriched in CaCO_3_ and are typically separated from the surrounding sedimentary rock matrices by sharp boundaries^1^,^2^,^3^. Many of them have various kinds of well-preserved fossils at their centres. Isotopic analyses of concretions can be used to understand the diagenetic processes during burial and concretion formation^4^,^5^,^6^ and δ^13^C data show that calcite precipitates prior to significant methanogenesis^7^,^8^. Even though there have been many studies of concretions over several decades^1^,^2^,^7^,^8^,^9^,^10^,^11^,^12^, the source of carbon in isolated concretions and the mass transport processes controlling their growth rates are still poorly understood. The formation of spherical concretions has been explained by diffusion accompanied by carbonate inter-conversion reactions^10^,^12^. However, previously proposed models cannot explain the steep chemical gradients (notably of Ca) that occur within a thin-layer across the margin of a concretion to the host sediments, and the small variability of calcite concentration^13^,^14^ and porosity^15^ within the body of a concretion.

## Results and Discussions

**Figure 1:**
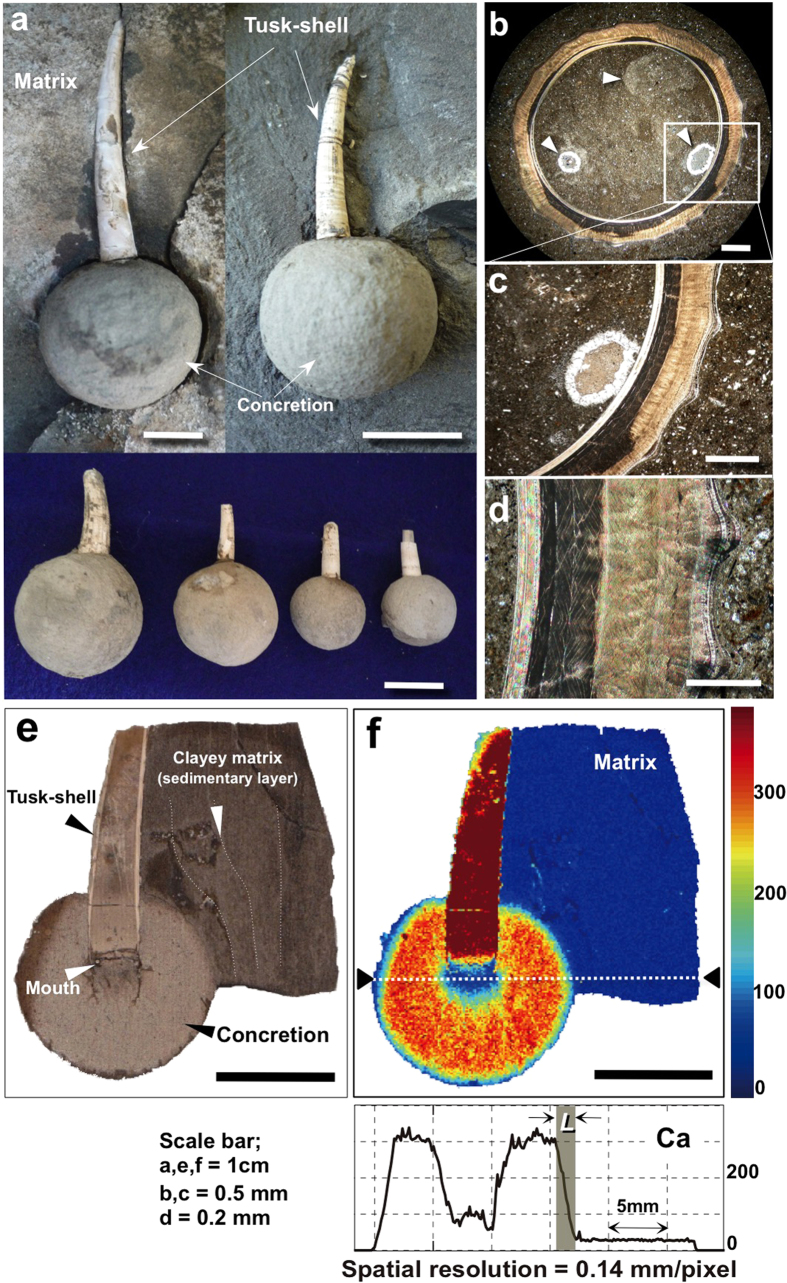
Occurrence of tusk-shell concretion. Spherical concretions formed around the mouth of a tusk-shell (*Fissidentalium* spp.)^17^ collected from the Kurosedani Formation of the Yatsuo Group distributed in Toyama Prefecture, Central Japan (Supplementary Fig. 1). (**a**) the diameter of the concretions is in the range 1 ~ 2 cm, with larger concretions occurring around larger tusk-shells. (**b,c**) cross section through a tusk-shell showing the internal texture, with porosity filled by precipitated carbonate (arrows in b). (**d**) aragonite layers forming a tusk-shell. (**e**) cross section showing a concretion formed around the mouth of a tusk-shell, which is in the centre of the picture. (**f**) SXAM Ca X-ray intensity in and around the cut surface of a concretion (**e**). A sharp boundary ‘L’ between the concretion and matrix is also defined by the Ca distribution. All photographs (**a–e**) shown here are taken by H.Yoshida.

**Figure 2:**
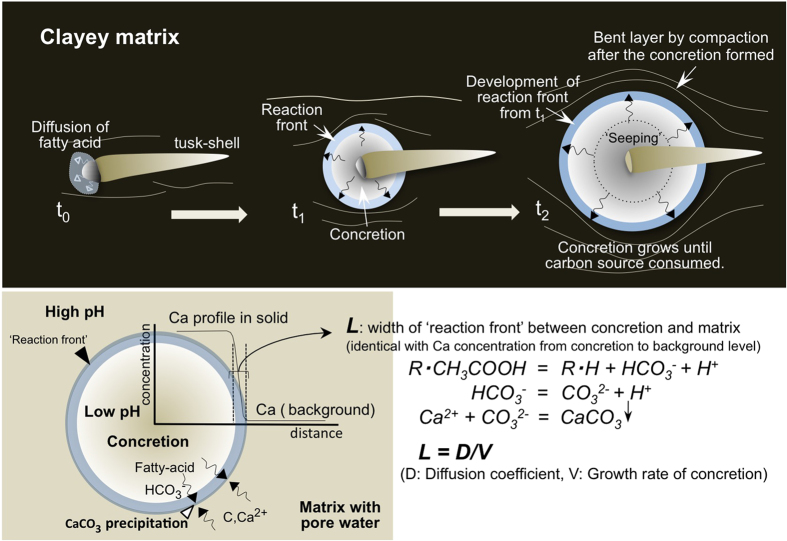
Isolated concretion formation mechanism. A spherical isolated concretion formed by the reaction between 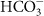, a byproduct from the breakdown of fatty acid originating from the dead organism in the tusk-shell, and pore-water Ca, which was mostly derived from the surrounding matrix outside the concretion. Precipitation of these species as CaCO_3_ occurred very rapidly and formed a hard concretion. The concretions continued to grow until the carbon of the organs was all consumed. Surrounding sedimentary layers have been bent by compaction after concretion formation was completed. The relationship between L (width of reaction front), D (diffusion coefficient of fatty acid in clayey matrix), and V (linear growth rate of reaction front) can be described as L = D/V.

Here we describe spherical carbonate concretions from a Neogene formation in the Yatsuo area of Toyama Prefecture, Japan (Fig. 1; Supplementary Fig. 1)^16^ and present evidence for the sources of their carbonate constituents, growth rates and formation conditions. The concretions are formed around tusk-shells (*Fissidentalium* spp.)^17^ buried in compacted clayey mudrock (ca. 50% of porosity; Table 1; Supplementary Fig. 2a–c and Table 1). The matrix has a high proportion of smectite (ca. 19 wt% of solids) compared with the concretion (ca. 5 wt% of solids) (Table 1 and Supplementary Fig. 2c and Fig. 2d). Well-rounded isolated concretions with diameters of less than a few centimetres (diameters of concretions φ : 14 ~ 37 mm), depending on the tusk-shell sizes (Supplementary Fig. 2a), were developed around the open ends of the tusk-shells, where most of the soft tissues of the animals would have been concentrated (Fig. 1a). The tusk-shells lie almost parallel to the sedimentary layers, in their life positions. Very fine sedimentary layers in the clayey matrix are deviated around the boundary of each concretion showing that the concretions grew at a relatively early stage of diagenesis, before sediment compaction by burial (Fig. 1e and Supplementary Fig. 2b). Detailed optical microscopic observations on thin-sections cut across the bodies of the tusk-shells and X-ray diffraction data from each component of the tusk-shell and surrounding rock matrix reveal that the tusk-shells are almost pure aragonite (Supplementary Fig. 3). In contrast a concretion only has calcite as the pore filling and formed by expansion into the surrounding sediment as a result of calcite deposition in the sediment’s pores; the pores in the surrounding matrix are not calcite-filled (Supplementary Fig. 2c,d). The internal textures of the tusk-shells are also still observable (Fig. 1b,d). The occurrence of spherical concretions and tusk-shells within homogenous clayey sediments shows that the spherical concretions formed in a system that can be treated as being closed. Almost no hydraulic gradient is expected in such fine seabed sediments and therefore any elemental mass transport was likely controlled by diffusion. This conclusion is supported by the spherical geometry of the concretions.

Aragonite preservation and the mineralogical characteristics imply that the tusk-shells have not been dissolved and the calcite (CaCO_3_) that composes each concretion was formed by precipitation of Ca supplied from the pore fluid within the buried sediment. δ^13^C values (**−**19.2 to **−**15.9‰) of the spherical concretion show that the carbon in the concretions is of biogenic origin^18^, but the aragonite that forms a tusk-shell has a δ^13^C value (+1.7 to + 2.3‰) consistent with that of marine carbonate (Table 1 and Supplementary Table 2). The carbon concentration in the concretion (6.1 ~ 8.9 wt%) is three orders of magnitude higher than that of the surrounding sedimentary matrix (0.01 wt%; Table 1). P concentration is elevated in the concretion and is consistent with an organic component being present (Supplementary Table 3). Based on the different Ca concentrations in the concretions and surrounding sediments (ca. 45 ~ 48 wt%; Supplementary Table 3) and assuming that all Ca originated in seawater (ca. 0.42 g/l), Ca mass balance based on present measured rock porosities indicates that the Ca of a concretion was concentrated from pore water within a distance 10–15 times greater than the radius of a concretion. The carbon concentration of a concretion is also consistent with the combination of carbon from seawater within the same pore volume^19^, and carbon from the decomposed organic matter in the tusk-shells, as estimated from measurements made on modern tusk-shells of similar size (Supplementary Table 2).

Due to calcite precipitation within the pore space, a concretion’s Ca concentration became greater than that of the matrix by a factor of at least 10. Mapping by scanning X-ray analytical microscope (SXAM; Methods) revealed that Ca concentrations vary little in the concretions, but showed contrasts in elemental concentrations across the concretion and matrix (Fig. 1f, Supplementary Table 3 and Fig. 4a–c). The boundary i.e. ‘reaction front’ between a concretion and the surrounding matrix is defined by a steep change of Ca concentration, from a higher and almost invariant concentration within the concretion to a much lower background level in the matrix. Such a steep Ca concentration profile shows that the width of a reaction front ‘L’ has been controlled by the diffusion rate of 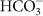, a byproduct of fatty acid (R-COOH) decomposition^20^,^21^, and rapid CaCO_3_ precipitation due to a pH change^20^ at the front (Fig. 2). During the reaction, both concentrations of 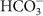 and Ca^2+^ in the pore-water were decreased at the reaction front, producing concentration gradients that drove further supply of these components to the reaction zone until all the 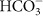 was consumed. That is, the concretions continued to grow until there was no more carbon of organic origin remaining. The reaction then stopped and the concentration of Ca^2+^ in pore-water returned to the same level as in seawater, so that the migration of Ca^2+^ also terminated. Micro-pores within the concretion functioned as migration paths for 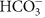 even after calcite precipitated at the reaction front. Calcite crystals grew preferentially within the initially relatively large pores in the matrix (Supplementary Fig. 2b) and continued until a balance was attained between the rates at which 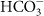 and Ca^2+^ were supplied by diffusion from opposite sides of the concretion’s boundary (controlled by pore geometry) and consumed by calcite precipitation in the thin region of the front. Complementary variations in Fe and Mn concentrations across a concretion are consistent with pH increasing from the centre towards the margin, since as pH decreases at a given redox potential Fe tends to partition into calcite preferentially with respect to Mn^22^ (Supplementary Fig. 4b,c).

Based on the evidence, here we propose a ‘seeping organic model’ that explains the formation of an ‘isolated’ concretion and the development of a ‘reaction front’, where CaCO_3_ precipitation proceeds. After the death of the organism, a tusk-shell is buried in the clayey sediment and filled with pore water containing Ca^2+^ ions. Decomposition of the dead body within the tusk-shell produces fatty acid, which seeps from the mouth of the tusk-shell and mixes with pore water that diffuses towards the shell from the surrounding sediment. The fatty acid decomposes during the mixing to give 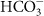 which then forms CO_3_^2−^ and reacts with Ca^2+^ in the pore water. Consequently, pH increases^20^ with increasing distance from the tusk-shell’s mouth (Fig. 2) leading to CaCO_3_ precipitation at the reaction front and hence an increase in the concretion’s diameter. The rate of calcite precipitation across the reaction front depends upon the activities of CO_3_^2-^, Ca^2+^, and H^+^ (i.e. pH)^23^. The diffusive fluxes of these chemical components depend upon both the concentration gradients across the concretion’s margin and the nature of the pores in the concretion and sediment. As the Ca^2+^ is supplied from the matrix pore-water outside the concretion, calcite precipitates until the porosity decreases to the observed value of ca. 25%. This process decreases the rate of diffusion towards the concretion’s boundary of 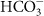 provided by the fatty acids, which is controlled by porosity and pore geometry (Fig. 2). The volume of sediments that can be affected by the decay of an animal of a given volume depends on the decay rate of the organism and of the diffusion coefficient of the sediments. A balance between the rates of solute transport and precipitation causes formation of a CaCO_3_ concretion with a porosity of 25%. Inside the concretion, further precipitation does not proceed.

The fatty acid produced by decomposition of the dead soft body tissues diffuses outwards from the shell’s mouth, through the remaining pores inside the concretion. Concentration factors of C and Ca in the concretion compared with the matrix sediments are about 600 ~ 800 and 12 ~ 13 respectively (Supplementary Tables 2 and 3).

In order for the concretion to grow, the diffusion rate of fatty acid must be faster than the rate of Ca^2+^ diffusion inwards from the concretion’s rim. The reason is that the Ca-concentration gradient in pore-water is less steep than the organic acid concentration gradient. The relatively low pore-water Ca concentration gradient is caused by the Ca concentration in the concretion being buffered by calcite and the Ca concentration outside the concretion being controlled by the volumetrically large reservoir of pore-water. In contrast, the organic acid concentration in the surrounding pore-water is much lower than in the concretion, which is in close proximity to the decaying organism in the tusk-shell. An 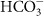 ion diffusing from the surface of the concretion precipitates within the width of the reaction front [L]. If the position of the reaction front does not move, L increases as 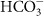 ions diffuse outward. However, the position of the front moves as the concretion grows outward.

Then the width of the reaction front [L] is therefore related to the diffusion coefficient [D] and the growth rate of the concretion [V = dR/dt (R: radius of a concretion, t: time)] by:


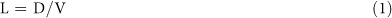


The growth rate V decreases as the concretion becomes bigger, owing to the 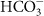 ion concentration gradient decreasing. From the presently observed chemical gradients around a concretion, we can determine L (~0.2 cm) (Eq. 1), corresponding to the final stage of concretion development. The final growth rate of the concretion can be used to estimate a maximum growth rate at the final stage, a growth timescale τ is given by:


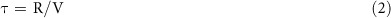


where R is the radius of a concretion.

**Figure 3:**
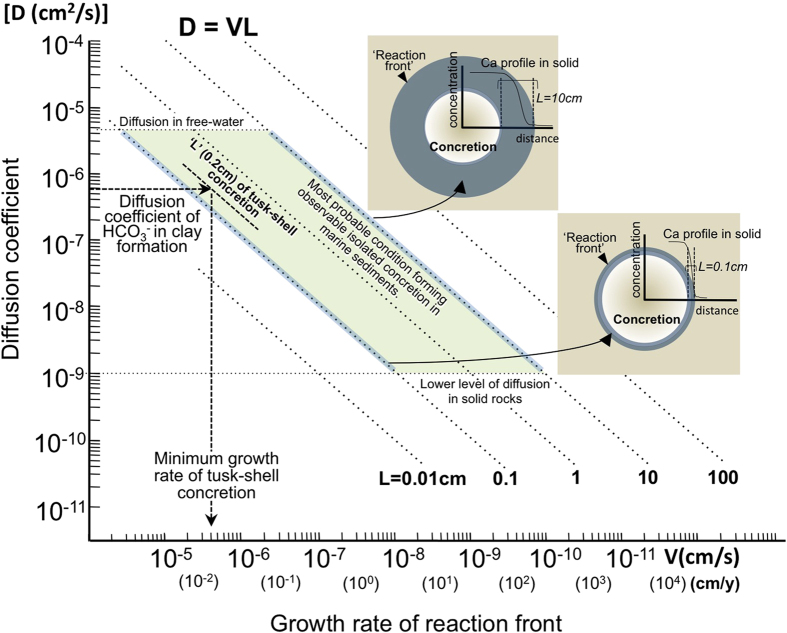
Diffusion–growth rate cross-plot. Relationship between effective diffusion coefficient (D; cm^2^/s) and growth rate of reaction front (V; cm/s) defined by dimension analysis. The field over which the tusk-shell concretions most likely formed is defined by the width of the reaction front (L = 0.2 cm) and the effective diffusion coefficient of 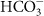 in similar kinds of clay formation (e.g. Boom Clay^24^,^25^). A very rapid minimum growth rate of the reaction front, in the order of 10^−6^ ~ 10^−5^ cm/s (weeks to months), is indicated.

From equations [1] and [2], we can constrain the diffusion rate of relevant ions through the sedimentary matrix, and the growth timescale of a concretion, as shown in a ‘Diffusion-growth rate cross-plot’ (Fig. 3). Published values of effective 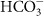 diffusion coefficients in Miocene clayey rocks with similar characteristics to those studied here (24,25; Supplementary Table 4), can be used to estimate the minimum growth rate (and hence the maximum growth time) of a concretion. This cross-plot also shows how the formation conditions of those kinds of isolated concretions that develop around localized reactive material in marine sediments can be estimated from the diffusion coefficients of these sediments and the widths of the reaction fronts at the margins of the concretions.

## Conclusion

In summary, the ‘seeping organic model’ described here and tested using measured values for ‘L’ and radii ‘R’ of concretions, readily explains the formation processes of the concretions and allows us to estimate concretion growth times (Fig. 3). In particular, parametric analysis of transport processes shows that a sharp interface is only developed when diffusion occurs in combination with relatively rapid carbonate mineral precipitation, such that concretions of the size examined may form within a time scale of weeks to months. This is also consistent with reported isolated concretion occurrences in unconsolidated Holocene sediments^26^. Although isolated spherical concretions do not always have visible fossils inside, the findings shown here suggest that concretions that are not associated with fossils might also be made from carbon sourced in the dead soft body tissues of organisms^27^ without skeletons.

## Methods

Geological and geometrical characterization of sediments and concretions (thin-section, SEM-EDS, XRD and porosity measurement), paleontological examination of tusk-shells and geochemical analysis (XRF) were carried out in the Nagoya University Museum and Graduate School of Environmental Science of Nagoya University.

Quantitative data for smectite in the sediment and concretion were obtained by analysis using the internal standard method at the Hokkaido Soil Research Cooperation, Japan. X-ray diffraction (XRD) peaks produced by pulverized samples were measured after cation exchange for Ca^2+^. Fluorite was used as the internal standard. The calibration curve was selected based on the integral width of the peak near 2θ = 6° (d = 15 Å) of the XRD pattern.

A secondary X-ray analytical microscopy (SXAM) method that uses an X-ray fluorescence analyzer (XGT-2000V Horiba Japan) to show semi-quantitatively the two-dimensional distribution of elements across the whole surface of a sample was employed at the Department of Education, Gifu University, Gifu, Japan. A high-intensity continuous X-ray beam (Rh anode 50 kV 1 mA), 100 μm in diameter, is focused with a guide tube and irradiated perpendicular to the surface of the sample, which is located on a PC-controllable X-Y stage. X-ray fluorescence from the sample surface is analyzed with the hp-Si detector of an energy-dispersion spectrometer.

The δ^13^C and carbon contents of pulverized carbonate samples were measured by placing each sample in a reaction container that had been purged by He gas and then reacting the sample with H_3_PO_4_ introduced by a syringe. After being reacted, the evolved CO_2_ gas was carried into an IR-MS (Thermo Fisher DELTA V Plus) in a He carrier gas to measure the carbon stable isotopic ratio (δ^13^C_V-PDB_).

All full description of the methodology applied in the present study is given in the Supplementary Information.

## Additional Information

**How to cite this article**: Yoshida, H. *et al.* Early post-mortem formation of carbonate concretions around tusk-shells over week-month timescales. *Sci. Rep.* **5**, 14123; doi: 10.1038/srep14123 (2015).

## Supplementary Material

Supplementary Information

## Figures and Tables

**Table 1:** Representative characteristics of a concretion and matrix.

	**Concretion**	**Matrix**
Porosity (%) Density (g/cm^3^) Smectite (wt%)	27.0 ~ 28.8 1.7 ~ 1.8 5.0	51.1 ~ 55.9 1.4 ~ 1.5 19.0
XRF analysis (wt%)* CaO EA method (wt%) C	45.7 ~ 52.1 6.07 ~ 8.86	3.8 ~ 4.0 0.01
_v-PDB_(‰)	−19.2 ~ −15.9 (tusk-shell + 1.7 ~ +2.3)	−8.3 ~ −6.4

Porosity, density, contents of clay minerals and element concentrations^*^ (CaO and C) and δ^13^C_v-PDB_. *All other major element concentrations are shown in Supplementary Tables 1 –3.

## References

[b1] Coleman M. L. Microbial processes: Controls on the shape and composition of carbonate concretions. Marine Geology 113, 127–140 (1993).

[b2] Mozley P. S. & Davis J. M. Internal structure and mode of growth of elongate calcite concretions: Evidence for small-scale, microbially induced, chemical heterogeneity in groundwater. GSA Bulletin 117, 1400–1412 (2005).

[b3] Dix G. R. & Mullins H. T. Shallow, Subsurface growth and burial alteration of Middle Devonian calcite concretions. Jour. Sedimentary Petology 57, 140–152 (1987).

[b4] Irwin H., Curtis C. & Coleman M. Isotopic evidence for source of diagenetic carbonates formed during burial of organic-rich sediments. Nature 269, 209–213 (1977).

[b5] Curtis C. D., Coleman M. L. & Love L. G. Pore water evolution during sediment burial from isotopic and mineral chemistry of calcite, dolomite and siderite concretions. Geochim. Cosmochim. Acta 50, 2321–2334 (1986).

[b6] Scotchman I. C. The geochemistry of concretions from the Kimmeridge Clay Formation of southern and eastern England. Sedimentology 38, 79–106 (1991).

[b7] Mozley P. & Burns S. J. Oxygen and carbon isotopic composition of marine carbonate concretions: An overview. J. Sedim. Petrol. 63, 73–83 (1993).

[b8] Raiswell R. & Fisher Q. J. Mudrock-hosted carbonate concretions: a review of growth mechanisms and their influence on chemical and isotopic composition. Jour. Geological Society, London 157, 239–251 (2000).

[b9] Berner R. A. Rate of concretion growth. Geochim. Cosmochim. Acta 32, 477–483 (1968).

[b10] Wilkinson M. & Damper M. D. The rate of growth of sandstone-hosted calcite concretions. Geochim. Cosmochim. Acta 54, 3391–3399 (1990).

[b11] Raiswell R. Non-steady state microbiological diagenesis and the origin of concretions and nodular limestones. in Diagenesis of Sedimentary Sequences (ed. Marshall J. D.) Geological Society Special Publication, 41–54 (1987).

[b12] Berner R. A. in Early Diagenesis, a Theoretical Approach, Ch.3, Diagenetic Physical and Biological Processes, 15–55 (Princeton University Press, 1980).

[b13] Raiswell R. The microbiological formation of carbonate concretions in the Upper Lias of NE England. Chemical Geology, 18, 227–244 (1976).

[b14] Coleman M. L. & Raiswell R. Carbon, oxygen and sulphur isotope variations in concretions from the Upper Lias of NE England. Geochim. Cosmochim. Acta 45, 329–340 (1981).

[b15] Raiswell R. The growth of Cambrian and Liassic Concretions. Sedimentology, 17, 147–171 (1971).

[b16] Hayakawa H. & Takemura A. The neogene system in the Yatsuo area, Toyama Prefecture, central Japan. Jour. Geol. Soc. Japan 98, 717–732 (1987).

[b17] Lamprell K. L. & Healy J. M. A revision of the Scaphopoda from Australian waters (Mollusca). Records of the Australian Museum, Supplement 24, 1–189 (1998).

[b18] Schidlowski *et al.* in Earth’s Earliest Biosphere: Its Origin and Evolution (ed. J. W. Schopf), Ch.7, Isotopic inferences of ancient biochemistries: carbon, sulfur, hydrogen and nitrogen. 149–186 (Princeton University Press, New Jersey, 1983).

[b19] McCorkle D. C., Emerson S. R. & Quay P. Carbon isotopes in marine porewaters. Earth Planet Sci Lett 74, 13–26 (1985).

[b20] Berner R. A. Calcium carbonate concentrations formed by the decomposition of organic matter. Science 159, 195–197 (1968). 5634908 10.1126/science.159.3811.195

[b21] Coleman M. L. & Raiswell R. Source of carbonate and origin of zonation in pyritiferous carbonate concretions: evaluation of a dynamic model. American Journal of Science 295, 282–308 (1995).

[b22] Barnaby R. J. & Rimstidt J. D. Redox conditions of calcite cementation interpreted from Mn and Fe contents of authigenic calcites. Geol. Soc. Am. Bull. 101, 795–804 (1989).

[b23] Lebron I. & Surez D.L. Calcite nucleation and precipitation kinetics as affected by dissolved organic matter at 25 °C and pH > 7.5. Geochim. Cosmochim. Acta 60, 2765–2776 (1996).

[b24] Aertsens M. *et al.* Vertical distribution of H^14^CO_3_^−^ transport parameters in Boom Clay in the Mol-1 borehole4 (Mol, Belgium) and comparison with data from independent measurements. External Report SCK SEN-ER-66 (2010).

[b25] European Atomic Energy Community (EURATOM). Effects of cement on clay barrier performance – Phase II (ECOCLAY II). EC CONTRACT No FIKW-CT-2000-00028 Final Report (2003).

[b26] Allison P. A. & Pye K. Early diagenetic mineralization and fossil preservation in modern carbonate concretions. PALAIOS 9, 561–575 (1994).

[b27] Lisa E. P. Geochemical and paleoenvironmental analysis of Lacustrine Anthropod-bearing concretions of the Barstow Formation, southern California. PALAIOS, 10, 44–57 (1995).

