# Supplementary material for: Early post-mortem formation of carbonate concretions around tusk-shells over week-month timescales
Source: Sci Rep. 2015 Sep 15;5:14123. doi: 10.1038/srep14123 (PMC4569893; doi:10.1038/srep14123)
Supplement: Supplementary Information [file srep14123-s1.pdf]

Early post-mortem formation of carbonate concretions around tusk-shells over week-month timescales

Supplementary data

Hidekazu Yoshida[1*], Atsushi Ujihara[2], Masayo Minami[3], Yoshihiro Asahara[2], Nagayoshi Katsuta[4], Koshi Yamamoto[2], Sin-iti Sirono[2], Ippei Maruyama[2], Shoji Nishimoto[5], Richard Metcalfe[6]

1) Material Research Section, Nagoya University, University Museum, Chikusa, Nagoya, Japan
*corresponding author) dora@num.nagoya-u.ac.jp
2) Graduate School of Environmental Studies, Nagoya University, Chikusa, Nagoya, Japan
3) Center for Chronological Research, Nagoya University, Chikusa, Nagoya, Japan
4) Department of Education, Gifu University, Gifu, Japan
5) Nagoya City Science Museum, Sakae, Nagoya, Japan
6) Quintessa UK, The Hub, Henley-on-Thames, Oxfordshire, UK

Geology and occurrence of tusk-shell concretions

Concretions around tusk-shells are found in the Kurosedani Formation of the Yatsuo Group, which is distributed in Toyama Prefecture, Central Japan (Supplementary Fig.1a). The Kurosedani Formation consists of argillaceous sedimentary rocks that have been determined

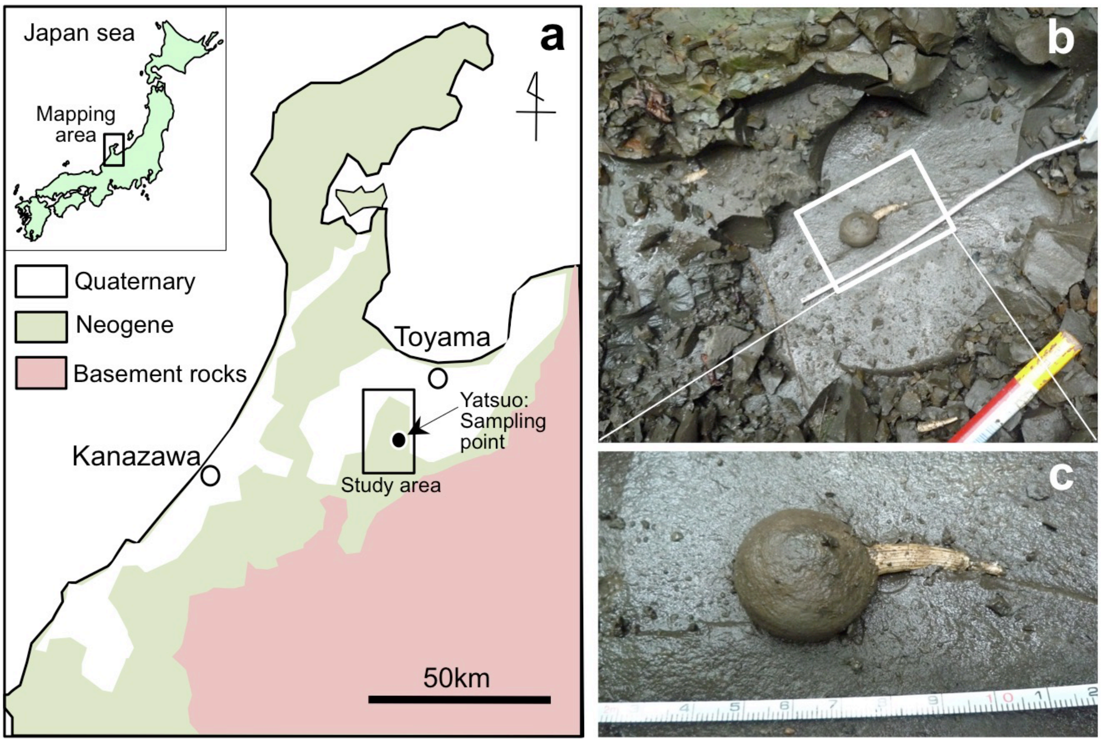

Supplementary Figure 1 Location and occurrence of tusk-shell
a, geological map of Yatsuo area. b,c, occurrence of tusk-shell at the outcrop. All tusk-shells lie almost parallel to the sedimentary layer. Geological map created by ppt based on the geological survey data.

to be of Miocene age (16 – 15 Ma) using diatoms and planktonic foraminifera (28), and magneto-stratigraphy (29). The total thickness of the Kurosedani Formation is ca. 500 m and tusk-shell concretions are observed in a clayey layer (about 20 metres thick) in the upper level of the formation. The occurrence of tusk-shells in outcrops is shown in Figures 1b and c, in which the shells can be seen to lie in the compacted clayey matrix almost parallel to the sedimentary layers. Based on the morphology and the shell surface texture, collected tusk-shells with concretion were determined to be *Fissidentalium* spp. (30).

Methodology and results of each geological and geochemical analysis

- **Size distribution analysis of concretion**

In order to identify the relationship between concretion size and tusk-shell size, *D* (average diameter of concretion) and *d* (diameter of tusk-shell mouth) of 24 well-preserved concretions with tusk-shells were measured across cut surfaces through the concretions. The results (Supplementary Fig. 2a) show a linear correlation between the concretion diameter and the size of tusk-shell mouth. This suggests that the carbonate source of the concretion is mainly from the decomposed organs of the tusk-shell.

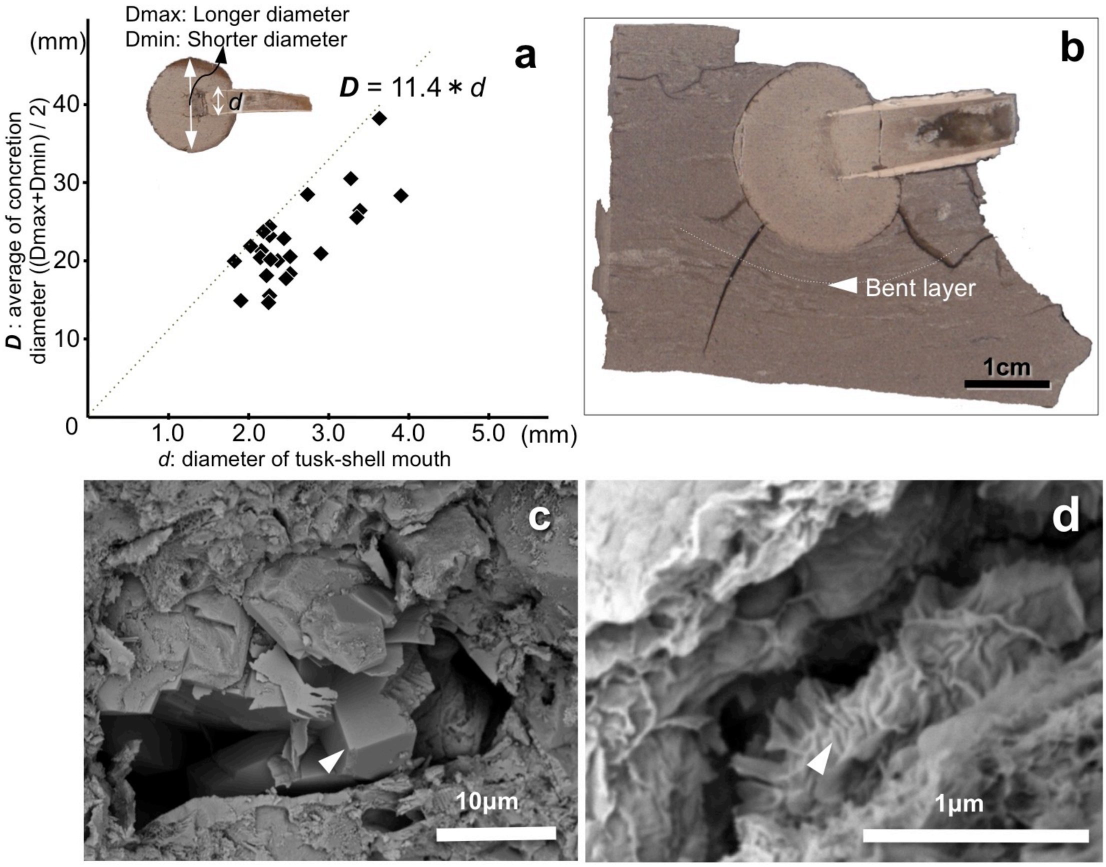

Supplementary Figure 2 Size distribution and internal texture of tusk-shell a, relationship between average diameter (Dmax: Longer diameter; Dmin: Shorter diameter) of concretion and diameter of tusk-shell showing a linear relation of concretion formation. b, cross-cut photo of tusk-shell with layer bent around the concretion. SEM photomicrograph of the pore texture observed in concretion with calcite crystal (c; arrow) and surrounding matrix with clay filling (c; arrow).

- **Microscopic observations**

Photomicrograph of a section through a concretion and tusk-shell (Supplementary Fig. 2b) shows clearly that sedimentary layers bend around the tusk-shell, which lays almost parallel to the sedimentary plane. SEM images show the difference between micro-pore filling minerals in the concretion and surrounding rock matrices, i.e. the micro-pores in a concretion's matrix are mainly filled by calcite micro-crystals (Supplementary Fig. 2c), whereas in contrast the pores of the surrounding matrix are clay-filled (Supplementary Fig. 2d).

- **Porosity measurement (Supplementary Table 1)**

Upon sampling, the rock samples and concretions were packed in plastic bags to prevent them from drying and being damaged during transportation to the laboratory. Immediately after arrival at the laboratory, the specimens were saturated with distilled water for 24 hours using a decompression chamber. After saturation, the weights (Ws) and volumes (V) were measured. The saturated specimens were then dried for 3 days at a temperature of 105 °C and then their dry weights were measured (Wd). As *Ws-Wd* corresponds approximately to void volume, the effective porosity was derived as follows (31):

$$\frac{Ws - Wd}{V} \times 100 \quad = \text{effective porosity (\%)}$$

The results show a clear volumetric difference between the concretion and the surrounding rock matrices due to the micro-pore sealing by calcite.

Supplementary Table 1 Geometrical features of the concretion and matrix.

	Weight (wet;g)	Weight (dry;g)	Volume (cm^3)	Porosity (%) & (Density g/cm^3)
**Concretion**				
1	15.29	13.13	7.5	28.8 (1.75)
2	18.98	16.48	9.0	27.8 (1.83)
3	20.49	17.79	10.0	27.0 (1.78)
4	22.45	19.40	11.2	27.2 (1.73)
**Matrix**				
5	51.53	36.72	26.5	55.9 (1.39)
6	52.37	38.31	27.5	51.1 (1.39)
7	85.66	62.84	42.6	53.5 (1.48)

- **XRD analysis**

Mineralogical compositions were determined with an X-ray diffractometer (XRD; Multiflex, Rigaku Co.) using crushed and powdered samples and Cu Kα radiation (the Cu being subjected to an electron beam of 40 kV/ 20 mA) (Supplementary Fig. 3). XRD patterns show clear differences between the calcite contents of the concretions and surrounding matrix. Quantitative analysis of smectite was also carried out by the internal standard method at the Hokkaido Soil Research Cooperation.

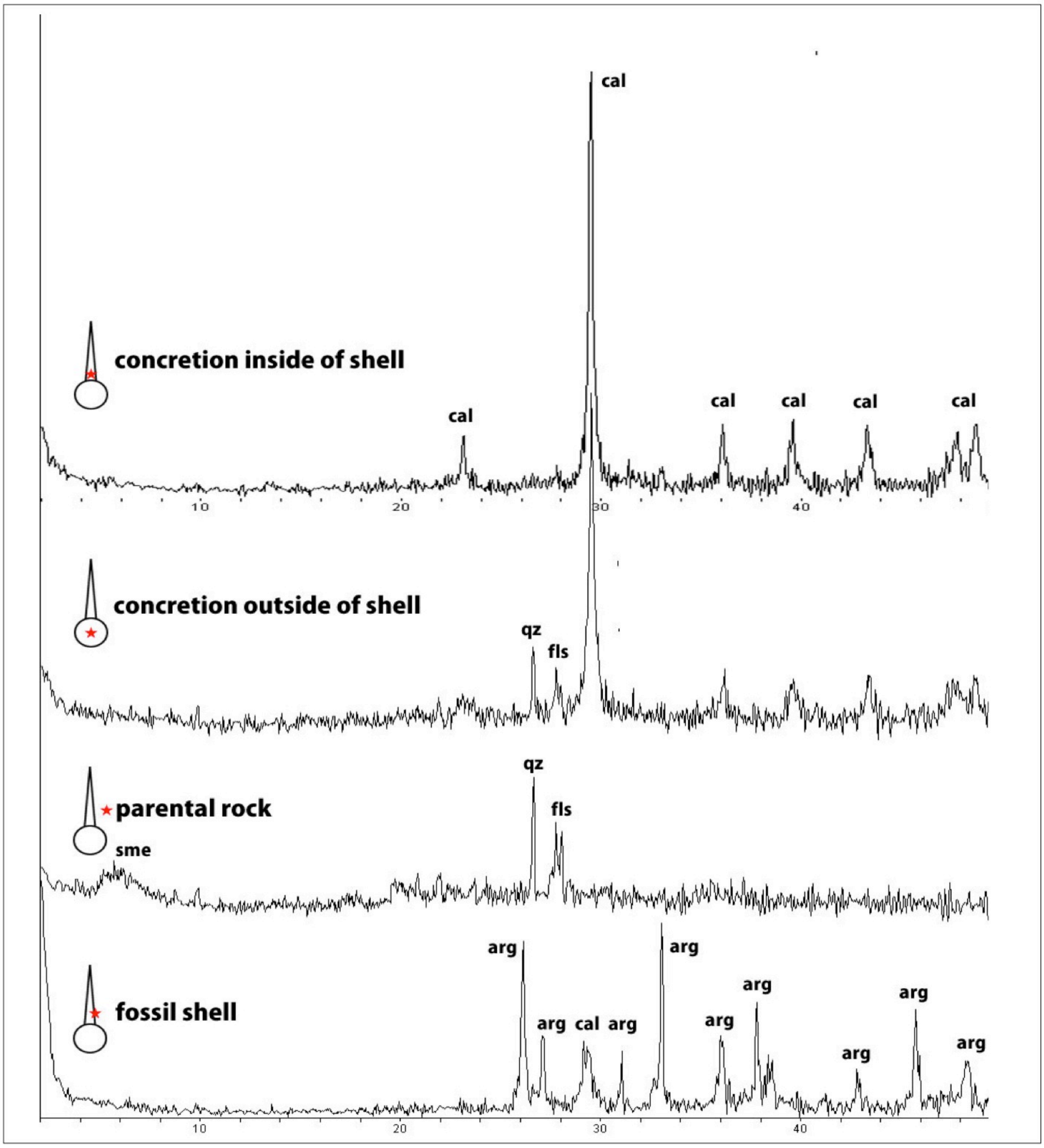

Supplementary Figure 3 XRD patterns Clear mineral differences composing in and around the concretion. cal: calcite, qz: quartz, fls: feldspar, arg: aragonite

- **SXAM analysis**

The SXAM intensity maps were reduced to one-dimensional element profiles in a direction perpendicular to the concentric ring pattern identified in a Ca map of a carbonate concretion (Supplementary Figs. 4a ~ c), using the lamination trace technique (32). Ca concentrations were found to be similar across a broad width of a concretion, but were relatively low in the volume originally occupied by the organism and decreased rapidly across the rim of the concretion (Supplementary Fig. 4a). Fe and Mn concentrations vary in a complementary fashion. The Fe concentrations are elevated in the volume originally occupied

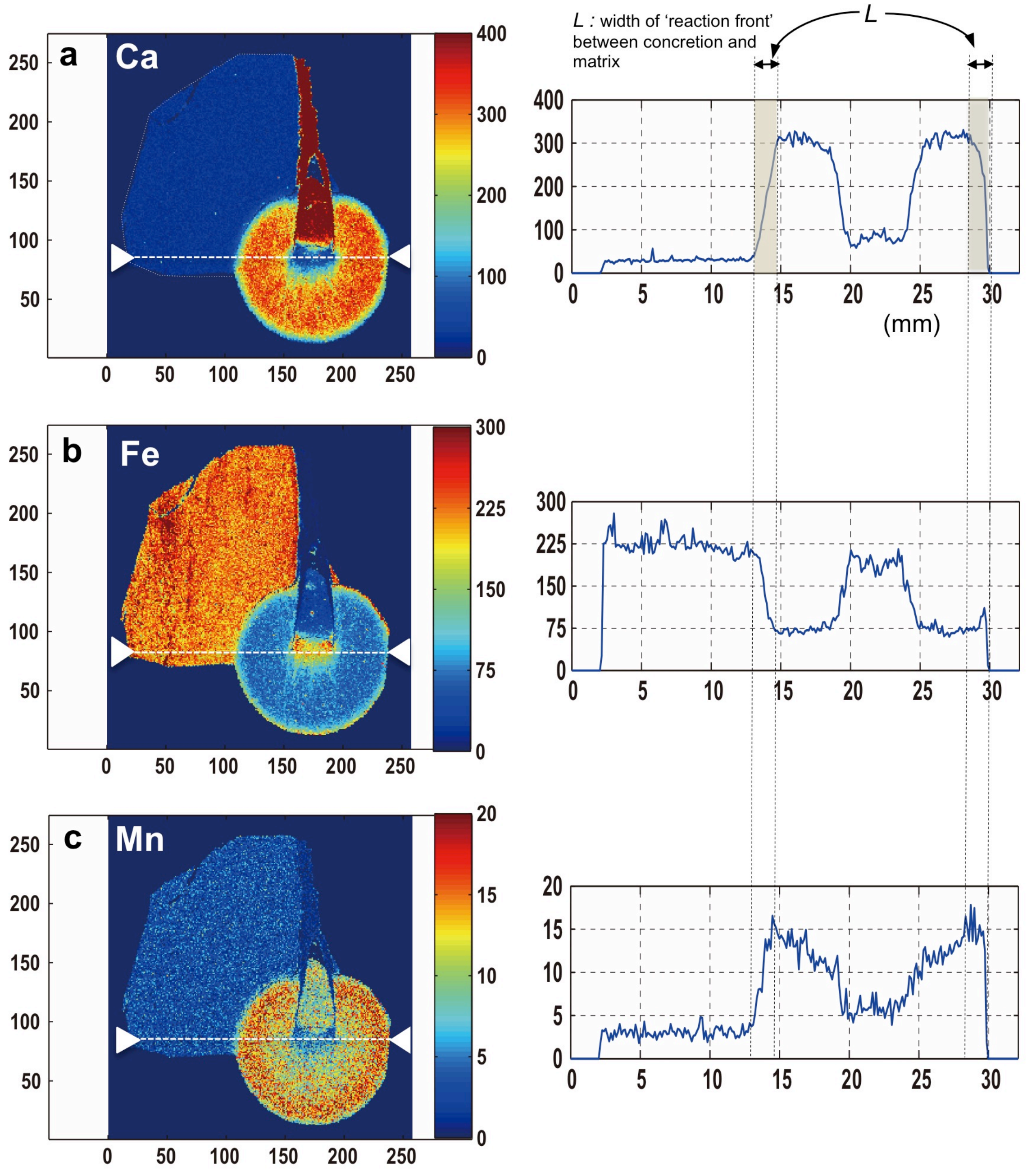

Supplementary Figure 4 Elements distribution in and around concretion Distribution of Ca, Fe and Mn have close correlation showing the influence of pH changes across the concretion to matrix.

by the organism, but Mn concentrations are relatively low in this zone. Outside this zone, Fe concentrations are relatively low, but Mn concentrations increase towards the margin of the concretion. This variation could be explained by the conditions towards the margin of the concretion being less reducing and more alkaline than those in the vicinity of the decaying organism; the stability field of Mn-carbonate extends to higher pH and Eh than the stability field of Fe-carbonate (33).

- **$\delta^{13}C$ and carbon contents measurement**

$\delta^{13}C$ and carbon contents of the internal organs of a tusk-shell were measured using EA-IRMS (Thermo Fisher DELTA V Advantage ConFloIV, coupled to an elemental analyzer FlashEA1112). The error of a $\delta^{13}C$ measurement is 0.1‰. All measured data are shown in Supplementary Table 2. In particular, the spatial coincidence of both the low $\delta^{13}C$ from the concretion and the original location of the organism shows that the carbon of the concretion is mainly from the tusk-shell.

Supplementary Table 2
Results of carbon contents and $\delta^{13}C$ measurement

		C (μg)	$\delta^{13}C$ vs PDB (‰)
**Fossil** (see text Figure 1)			
concretion	1	32.5 (7.20%)	-19.2
concretion	2	36.4 (7.87%)	-19.1
concretion	3	50.6 (8.36%)	-18.3
concretion	4	30.6 (6.07%)	-15.9
concretion	5	30.7 (7.54%)	-18.7
concretion	6	45.0 (8.86%)	-19.0
concretion	7	27.5 (6.20%)	-17.6
matrix	8	10.8 (0.01%)	-6.35
matrix	9	7.2 (0.01%)	-7.58
matrix	10	9.9 (0.01%)	-8.29
tusk-shell	11	83.0 (12.5%)	+1.13
tusk-shell	12	63.0 (13.6%)	+1.46
tusk-shell	13	62.0 (12.2%)	+1.64
**Living tusk-shell**			
shell	14	51.9 (12.6%)	+1.21
shell	15	48.8 (13.4%)	+1.25
shell	16	63.6 (14.2%)	+1.34
organ	17	40.7%	-15.9
organ	18	34.6%	-13.2
organ	19	34.9%	-14.7

- **XRF analysis**

These analyses were undertaken using a Shimadzu SXF-1200 equipped with a Rh X-ray tube. Glass beads were prepared by mixing a portion of each sample, which was ignited at 950°C to decomposed carbonates, with anhydrous lithium tetraborate flux and then fusing (34). Sample: flux ratios were 0.7g: 6.0g and 2.0g: 4.0g, for major and minor element analyses respectively. Measurements were calibrated with rock reference samples issued by the Geological Survey of Japan (GSJ: Geochemical Reference Sample Data Base, http://www.aist.co.jp/RIODB/db012/ welcome.html). Supplementary Table 3 shows the major element composition of a concretion (01 ~ 05) and surrounding matrix (06 ~ 09). A concretion has a Ca^{2+} content that is quite high compared to the matrix. The large ignition loss is consistent with the high content of clay (smectite) in the concretion, a feature that is consistent with the microscopic observation. All measured data are shown in Supplementary Table 3.

Supplementary Table 3 Results of XRF analysis

	01	02	03 Concretion	04	05	06	07 Matrix	08
SiO_2	28.79	34.03	31.64	28.05	33.83	62.54	62.14	60.43
TiO_2	0.25	0.32	0.29	0.24	0.32	0.92	0.90	0.90
Al_2O_3	6.68	8.15	7.43	6.46	8.08	17.79	17.51	17.14
Fe_2O_3	2.21	2.55	2.48	2.15	2.49	6.90	7.36	6.95
MnO	0.63	0.42	0.43	0.69	0.44	0.03	0.03	0.03
MgO	2.93	3.01	3.08	2.85	2.98	2.43	2.42	2.34
CaO	51.67	45.71	48.34	52.12	46.01	3.99	3.84	4.04
Na_2O	0.70	0.90	0.81	0.69	0.90	1.86	1.92	1.95
K_2O	0.67	0.89	0.80	0.64	0.89	1.79	1.78	1.75
P_2O_5	2.39	1.08	1.15	2.96	1.07	0.13	0.13	0.13
Total(wt%)	96.91	97.07	96.45	96.84	97.00	98.38	98.03	95.65
Ig.Loss (wt%)	32.34	30.63	31.51	32.62	30.52	12.12	12.30	12.05

- **Diffusion coefficient of similar type of clay stone (Boom Clay)**

Table 4 summarizes diffusion coefficients determined by in-situ measurement and laboratory percolation experiments on similar clay-stone i.e. Boom Clay, a Tertiary clay formation distributed in parts of western Europe (24, 25; references shown in the main text). These are values for somewhat consolidated, plastic clay sediments ("stiff clay") and can be used to estimate the minimum growth rate of a concretion.

Supplementary Table 4 Diffusion coefficient estimated from diffusion experiments with similar type of clay-stone (Boom Clay) .

	Diffusion coefficient (cm^2/s)	
$H^{14}CO_3^-$	$(6 \pm 3) \times 10^{-7}$	[Average diffusion coefficient of Boom Clay determined by in-situ measurement (24).]
	7.1×10^{-7}	[Diffusion coefficient of Boom Clay determined by percolation experiments (25).]

References

28) Yanagisawa, Y. Diatom biostratigraphy of the lower to middle Miocene sequence in the Yatsuo area, Toyama Prefecture, central Japan. *Bull. Geol. Surv. Japan*, **50**, 139-165 (1999).

29) Itoh, Y., Yanagisawa, Y. & Watanabe, M. Magnetostratigraphy and diatom biostratigraphy of Neogene rocks distributed in the Yatsuo area, central Japan. *Bull. Geol. Surv. Japan,* **50**, 215-223 (1999).
30) Shimek, R. & Steiner, G. Scaphopoda. Microscopic anatomy of invertebrates (edited by Harrison, F. W. & Kohn, A. J.), volume **6B**: *Mollusca II*, 719-781 (1997).
31) Gillham, R. W. & Cherry, J. A. Contaminant migration in saturated unconsolidated geologic deposits. in *Recent Trends in Hydrology* (ed. by T.N.Narashimham), *The Geological Society of America, Special Paper* **189**, 31-62 (1982).
32) Katsuta, N., Takano, M., Okaniwa, T. & Kumazawa, M. Image processing to extract sequential profiles with high spatial resolution from the 2D map of deformed laminated patterns. *Computers & Geosciences* **29**, 725–740 (2003).
33) Barnaby, R. J. & Rimstidt, J.D. Redox conditions of calcite cementation interpreted from Mn and Fe contents of authigenic calcites. *Geol. Soc. Am. Bull.* **101**, 795–804 (1989).
34) Yamamoto, K. & Morishita, T. Preparation of standard composites for the trace element analysis by X-ray fluorescene. *Jour. Geol. Soc. Japan* **103**, 1037-1045 (1997).
